# Photoacoustic imaging of peripheral vessels in extremities by large-scale synthetic matrix array

**DOI:** 10.1117/1.JBO.29.S1.S11519

**Published:** 2024-01-20

**Authors:** Shuang Li, Guangjie Zhang, Yibing Wang, Wenzhao Li, Yu Sun, Changhui Li

**Affiliations:** a Peking University, College of Future Technology, Department of Biomedical Engineering, Beijing, China; b Peking University, National Biomedical Imaging Center, Beijing, China

**Keywords:** peripheral vascular disease, photoacoustic imaging, synthetic matrix array, three-dimensional imaging

## Abstract

**Significance:**

Various peripheral vascular diseases (PVD) in extremities, such as arterial atherosclerosis or venous occlusion in arm or legs, are a serious global health threat. Noninvasive vascular imaging is of great value for both diagnosis and assessment of PVD.

**Approach:**

By scanning a one-dimensional non-focusing linear array, an equivalent large two-dimensional (2D) matrix array with hundreds of thousands or more ultrasound elements is formed, thereby achieving a wide signal reception angle as well as large imaging area for three-dimensional (3D) imaging of peripheral extremities.

**Aim:**

To provide a feasible bedside and noninvasive imaging method for vascular imaging in extremities.

**Results:**

Our system can achieve high-quality photoacoustic (PA) peripheral vessel imaging. The 3D subcutaneous vascular imaging results of the palms and arms of healthy volunteers demonstrate the superior performance of the system.

**Conclusions:**

This work proposes a clinically oriented PA 3D subcutaneous vascular imaging system for human extremities. The system employs a synthetic matrix array via scanning a one-dimensional non-focusing linear probe, providing noninvasive, high-resolution, and high-contrast images of human extremities. It has potential application value in the diagnosis and monitoring of vascular diseases.

## 1 Introduction

Peripheral vascular disease (PVD), such as peripheral artery disease (PAD) and venous occlusion, has become a serious global health threat.^1^^,^^2^ For instance, it is estimated that there are 202 million PAD patients worldwide in 2010.^3^ Since PVD commonly occurs at extremities, noninvasive imaging of subcutaneous vessels in extremities is important for the diagnosis and assessment of PVD. Owing to superior capability in non-invasively imaging subcutaneous vessels without the need of contrast agent, photoacoustic (PA) imaging has gained much attention in study of various vascular-related diseases.^4^^–^^8^

Several groups have developed PA systems to image peripheral vessels in extremities, including linear probes,^9^^–^^17^ full-ring system,^18^^,^^19^ semi-ring system,^20^ planar 2-D array,^21^^–^^23^ and even more complicated semi-hemisphere two-dimensional (2D) array system.^24^^–^^28^ Based on those systems, the subcutaneous vasculature in human extremities, including palms, forearms, legs and foot, was successfully imaged. To form three-dimensional (3D) PA images, the handheld array and the arc-shaped array, whose elements are typically cylindrically focused, scan the targeted area and stacking multiple 2D B-scan results. The self-focusing characteristic in array elements of those systems leads to much lower resolution in the elevational direction than that of the lateral direction. On the other hand, although the semi-hemisphere array system can provide high quality 3D imaging within its field of view, resolution drops quickly in out-of-focus regime, limiting its imaging depth. Ideally, a PAI with large 2D matrix array can provide both isotropic spatial resolution and deep image depth. However, 2D matrix arrays are highly complex and expensive. Therefore, those reported planar 2D US matrix arrays all have very limited element numbers as well as small size. The small 2D array needs to employ scanning in multiple directions and image stitching techniques to achieve 3D imaging with a larger field of view. To address this dilemma, we previously proposed a synthetic matrix US array method via scanning a non-focus linear array for breast imaging.^29^ By scanning a one-dimensional (1D) nonfocusing linear array, an equivalent 2D array with a large size and a large number of elements can be obtained, which has a large acquisition angle and large imaging area at much lower cost and system complexity. In this work, we further implement this strategy to perform 3D PA imaging of limb blood vessels. Unlike our previous breast imaging work,^29^ here we used “reflection mode” PA imaging method. Imaging results of the palms and arms indicate that this method can provide high-quality human peripheral vascular images non-invasively and label-free, and thus, it has the potential in the diagnosis and monitoring of peripheral vascular diseases.

## 2 Methods and Materials

### 2.1 Design of 3D Photoacoustic Imaging System

**Fig. 1:**
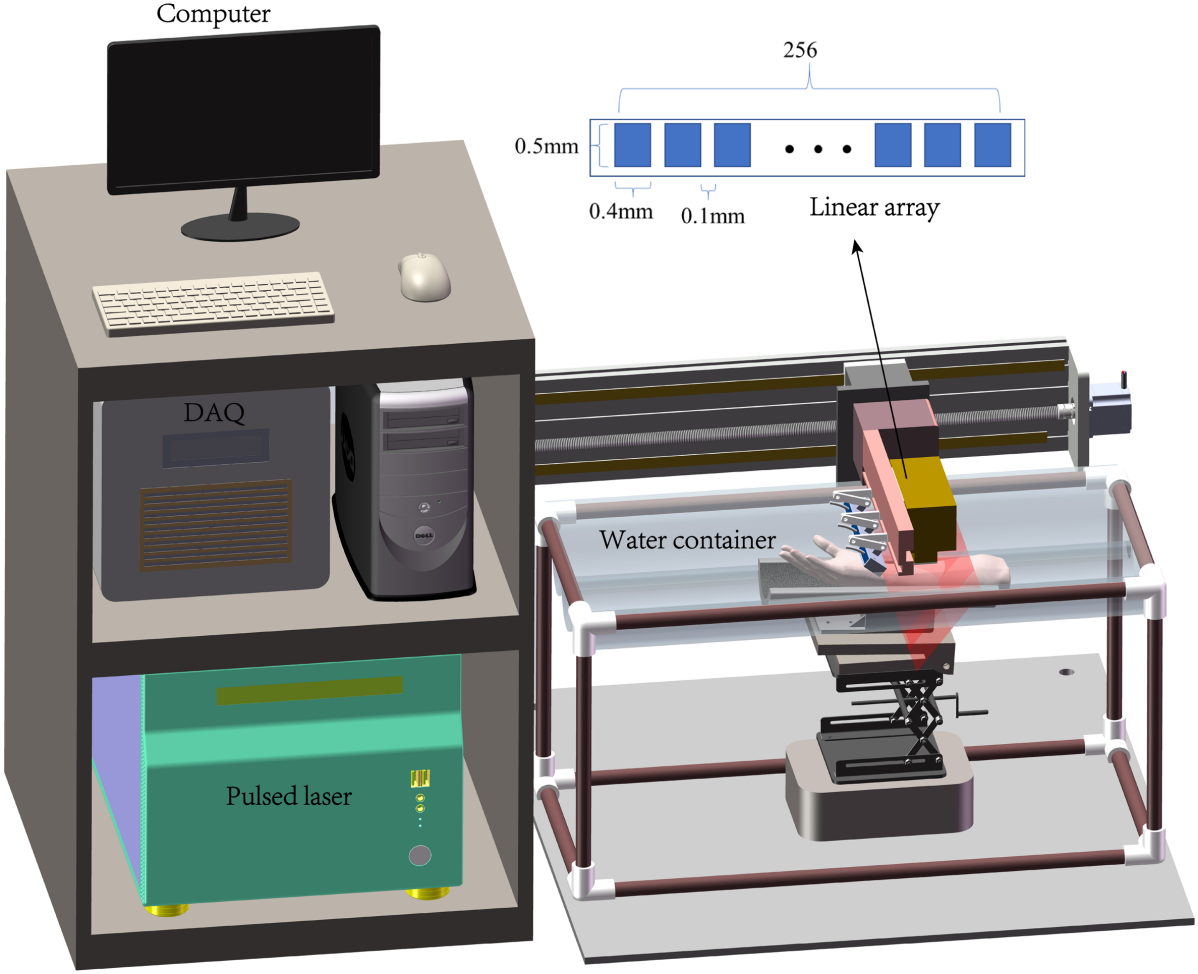
3D PA imaging system.

The proposed system uses a Q-switched Nd: YAG pulsed laser (LS-2137/2, LOTIS TII Ltd., Belarus) to excite PA signal. The wavelength is 1064 nm with a pulse width of ∼16  ns and a pulse repetition rate of 10 Hz. A customized one to three fiber bundle (Nanjing Chunhui Technology Industry Co., Ltd.) is used to deliver the laser. The system employed a nonfocusing linear array (customized by Imasonics, France) to receive PA signals. The linear array has 256 elements with a pitch of 0.5 mm and a kerf of 0.1 mm, i.e., a total length of 12.8 cm. The center frequency of the ultrasonic array is 3.5 MHz with over 80% bandwidth. PA signal is amplified 1500 times via self-built two-stage amplifier, and then received by a data acquisition system (Marsonics DAQ, Tianjin Langyuan Technology Co., Ltd. China) at 40 MHz sampling rate. The overall system setup is shown in Fig. 1. The unfocused linear array is mounted on a 1D electrical translational stage, which moves at a constant speed of 1  mm/s during imaging scanning. Three fiber bundle terminals are fixed on one side of the array to illuminate a large area of the imaged target.

The imaged objects, such as forearms, were immersed in a water tank made of soft PVC plastic film. There is a lifting device placed under the soft tank bottom, which can adjust the height and angle to support the imaged object, facilitating the top contour of the imaged body part approaching to the same horizontal level. Therefore, the distance between the probes to the object will not change much to maintain stable illumination condition, which helps the quantitative image reconstruction.

The excitation laser energy out of the fiber is about 195  mJ/pulse. The illumination area on the skin surface is approximately “14  cm×12  cm.” Therefore, the laser fluence on the skin is $1.2\;\mathrm{mJ}/{\mathrm{cm}}^{2}$, which is far below the safety threshold standard of $100\;\mathrm{mJ}/{\mathrm{cm}}^{2}$ at 1064 nm.^30^ It is worthy to note that although the illuminated area moves together with the ultrasonic array during scanning, the wide illumination condition still allows detection of PA signal from wide angle.

### 2.2 Illumination Strategy

**Fig. 2:**
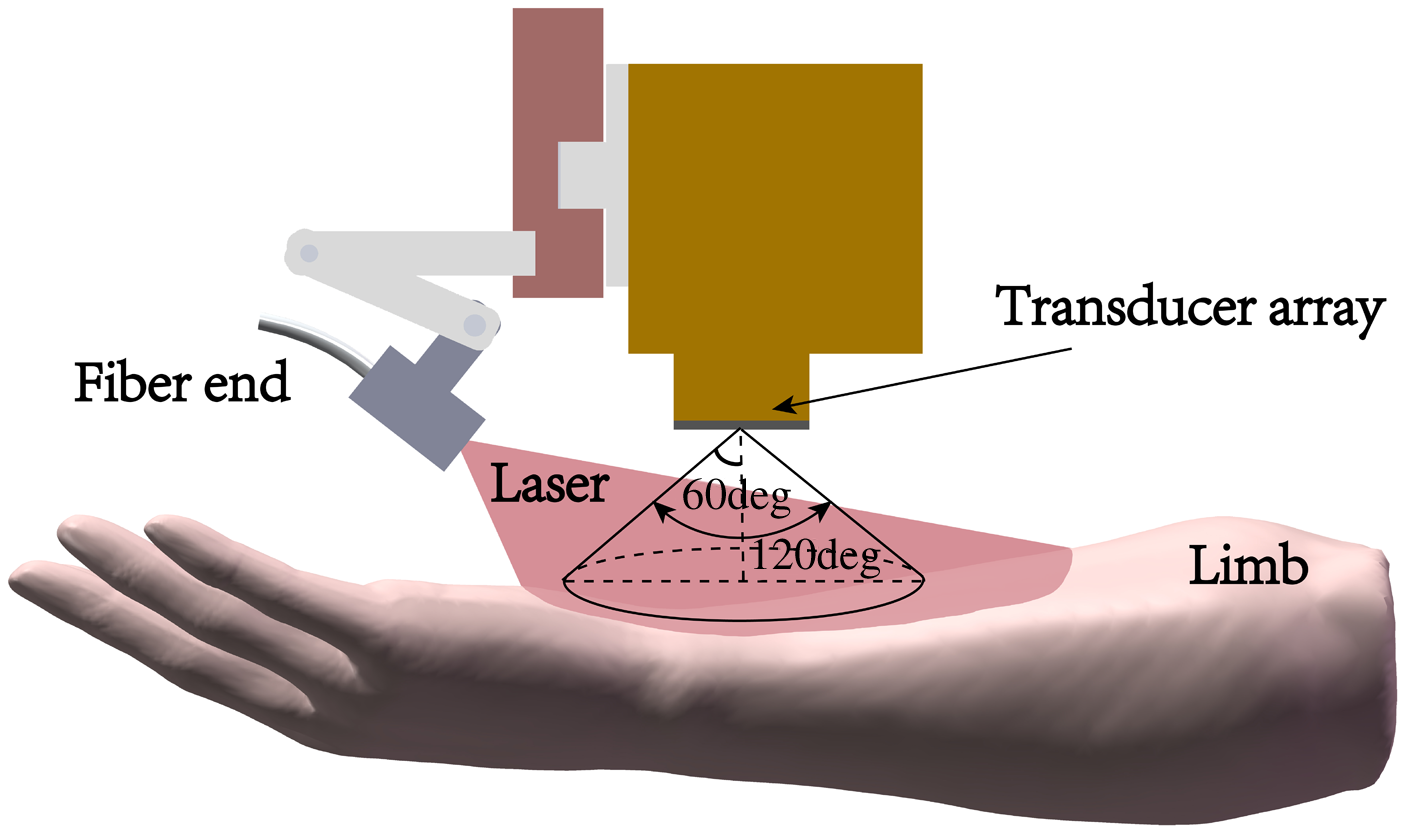
Schematic diagram of fiber optic illumination and one-dimensional array.

Compared to a focused linear array, our nonfocused linear array has a larger acceptance angle, allowing it to receive a broader range of PA signals. During the reconstruction process, we set a constraint condition that the relative angle between the directions of detector to the reconstruction point and normal of the detector to be less than 60 deg (Fig. 2). This requirement mandates that, during the scanning process, the dynamic illuminated area must cover a region over 60 degrees relative to the current array elements. Considering the numerical aperture of the glass fiber optic we use is 0.57, in our experiments, we adjust the illumination angle to around 40 deg.

### 2.3 Reconstruction Algorithm

**Fig. 3:**
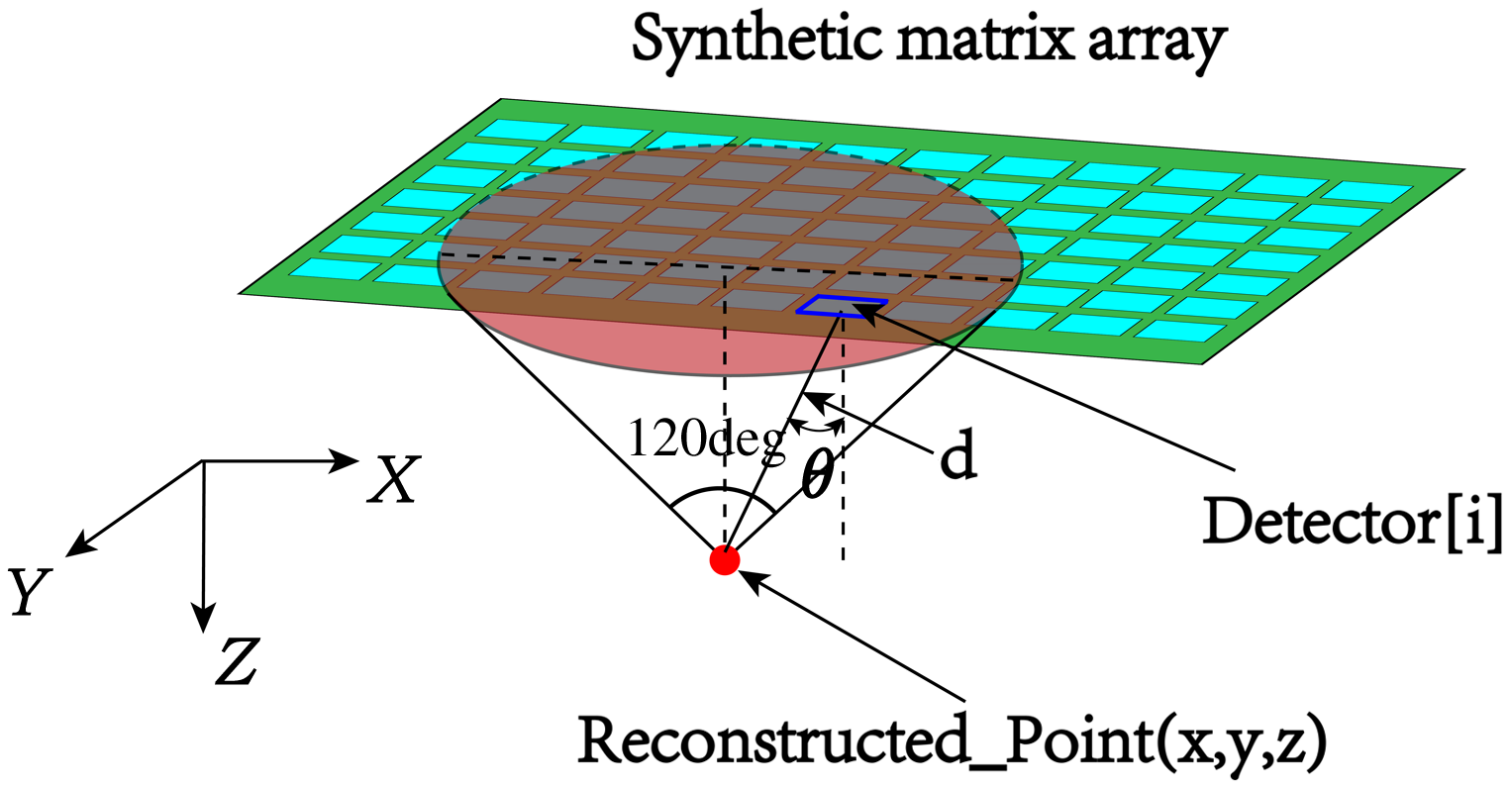
The back-projection algorithm with angular restriction for 3D PA imaging.

In the image reconstruction process, we employed the delay-and-sum (DAS) algorithm to reconstruct the image.^31^ Due to the moving finite size illumination condition, for each reconstruction point, as shown in Fig. 3, we only used PA data acquired by synthetic matrix array elements within a cone with a half aperture angle of 60 deg (θ is the angle between the line connecting the array element to the reconstructed point and the normal direction of the array element Detector[i]). The cone intersected with the plane containing the synthetic matrix forming a disk region (light red shaded area).

### 2.4 System Performance Testing

**Fig. 4:**
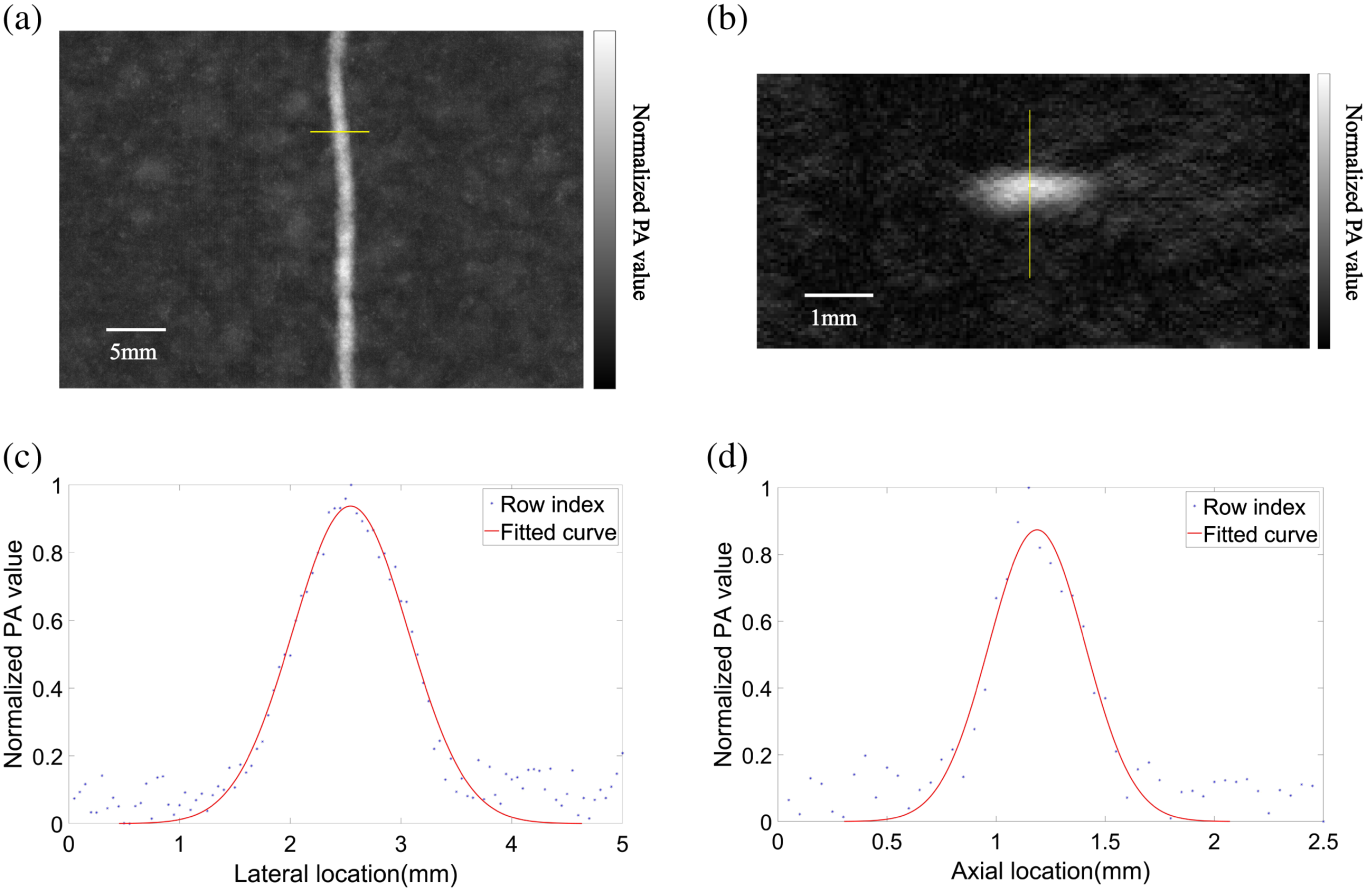
Resolution test. (a) MAP (scale: 5 mm). (b) Cross-section image along the marked line in panel (a) tomography image. (c) PA values and fitted curve fitted along yellow line in panel (a). (d) PA values and fitted curve along yellow line in panel (b).

The array probe has a length of 12.8 cm, scanning the array over a distance d can have an equivalent synthetic 2D matrix array with an area of 12.8  cm×d, and total elements are 256 × d/pitch. For instance, if d=10  cm, the total element number in this synthetic array is 53K. It is an enormous large-scale 2D matrix array. We have already presented an earlier version of this type of synthetic array to demonstrate its superior in acquisition angle and 3D imaging capabilities.^29^ So, here we only provide a resolution test result. A thin copper wire with a diameter of 0.1 mm is used, which is placed 19.2 mm below the array. Figure 4(a) showed the reconstructed maximum amplitude projection (MAP), as well as its cross-sectional B-scan result [Fig. 4(b)] image. Figures 4(c) and 4(d) plot the measured PA amplitude along lateral and axial directions, in which the yellow line is the fitted Gaussian curve. By calculating the full width at half maximum of fitted curves, the lateral and axial resolutions are of 1.23 and 0.52 mm, respectively. It is worth noting that due to the symmetric matrix structure, our system has the same lateral resolution as the elevational resolution, i.e., an isotropic spatial resolution.

## 3 Human Peripheral Vascular Imaging Results

We conducted 3D imaging of subcutaneous blood vessels in the human upper limbs of a healthy male adult volunteer. The human experiment in this study has passed the ethical approval of the Peking University Institutional Review Board.

**Fig. 5:**
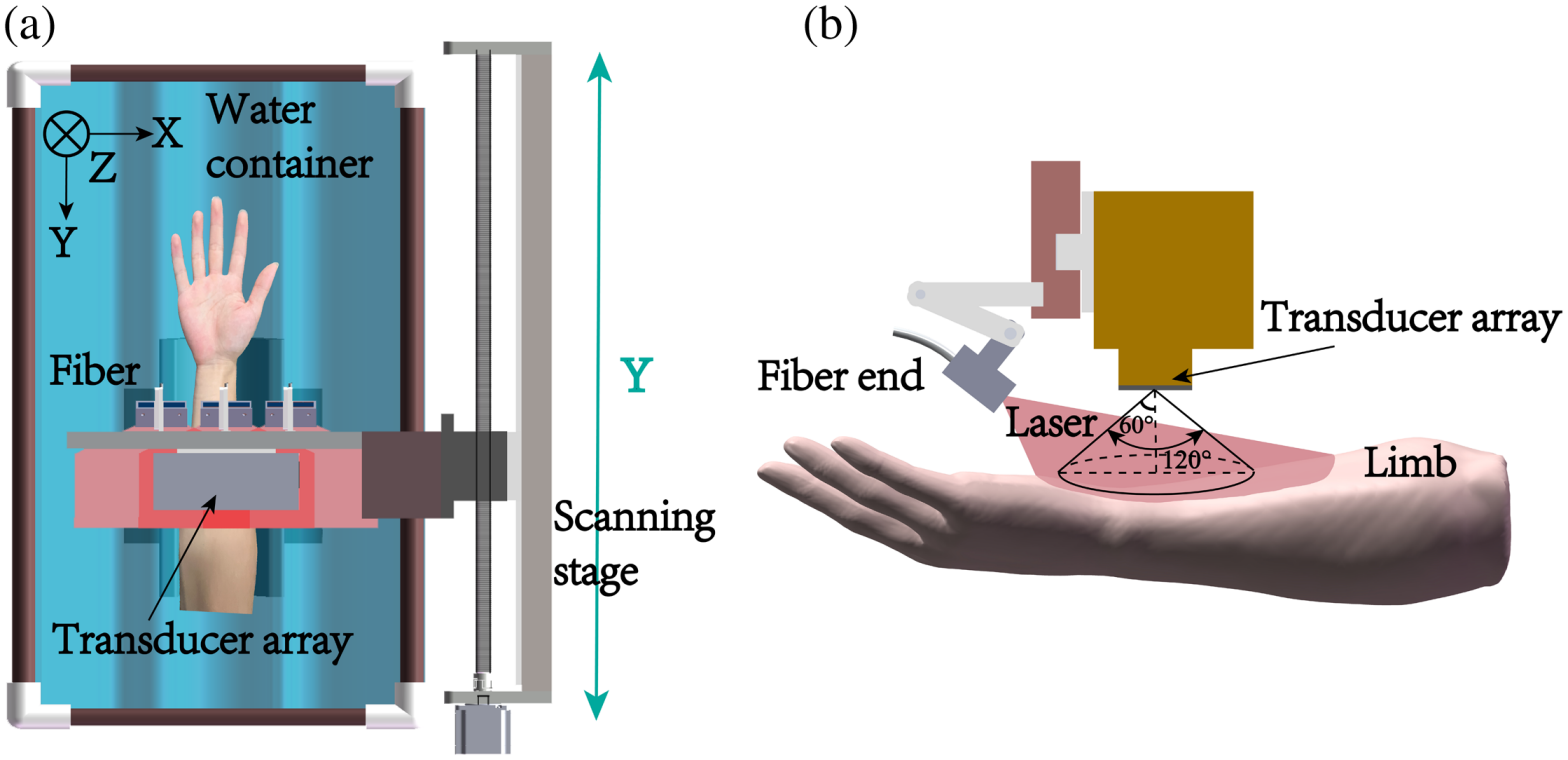
Schematic diagram of one-dimensional array scanning process. (a) The x direction is the array direction, the y direction is the scanning direction, and z direction is the depth direction. During the scanning process, both the arm and probe are in water, and red part represents the laser radiation area. (b) Schematic diagram of fiber optic illumination.

As aforementioned, to achieve relatively uniform illumination during the scanning process, the immersed arm is supported by an arc-shaped support and tilted to let the arm upper surface as horizontal as possible. As shown in Fig. 5, the 1D translational stage moves the linear array to scan over the limb at a scanning speed of 1  mm/s. The scanning length is scalable depending on study purposes, and both inner and outer arms, including palm, have been imaged.

**Fig. 6:**
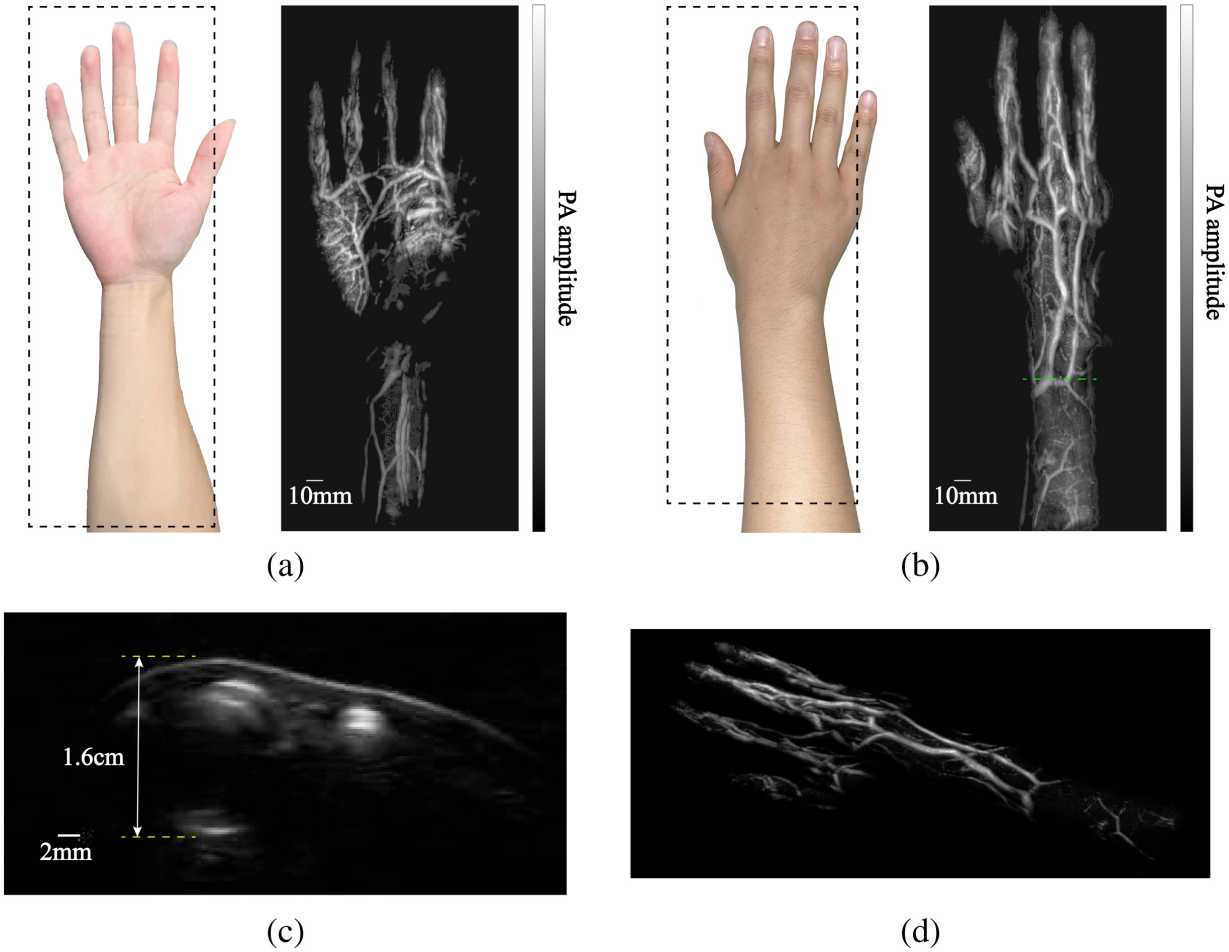
Imaging results of subcutaneous blood vessels in the human limb. (a) Photo of inner side of the right palm and forearm, and the MAP of 3D imaging of blood vessels of inner side of the right palm and forearm. (b) Photo of outer side of the right palm and forearm, and the maximum amplitude projection of 3D imaging of blood vessels of outer side of the right palm and forearm. (c) The cross-sectional B-scan image. (d) A snapshot of the 3D animation of the reconstructed vessels.

Figure 6 presents our 3D PA human imaging results, in which Figs. 6(a) and 6(b) are results of inner and outer sides of the right palm and forearm, respectively. The imaged area is marked by dashed boxes with a size of 140  mm×310  mm. It took 5 min to finish the scanning without signal averaging. Our results clearly show a rich subcutaneous blood vasculature with high contrast. Besides, the MAP results demonstrate isotropic lateral spatial resolution, which is a unique advantage of our synthetic matrix array. Figure 6(c) is the cross-sectional B-scan image over the dashed green line in Fig. 6(b). This B-scan image shows both skin and subcutaneous blood vessels at different depths. There is a deep vessel at 1.6 cm beneath the skin. Figure 6(d) is a snapshot of the 3D animation of the reconstructed vessels, the animation is available online (Video 1).

## 4 Conclusion and Discussion

In this work, we presented a 3D PA imaging system for peripheral subcutaneous vessels in extremities. Unlike those commonly used focused probes, our system employs a “synthetic matrix array,” which is realized by scanning a 1D unfocused linear array. With superior characteristics including wide acceptance angle, extreme large equivalent size and element number, this synthetic matrix array is suitable for PA imaging with large area and deeper depth. Our human studies demonstrated that abundant subcutaneous vascular structures in both inner and outer forearms and hands can been clearly displayed, and the current deepest vessel in this work locates at 1.6 cm beneath the skin. Besides, the manufacture technology to make 1D array is mature, our system substantially decreases in cost and complexity compared with a real large-scale 2D array. It is worthy to note that the synthetic matrix array features an adjustable scale, allowing for convenient modulation of the number of array elements by varying the scanning distance. This flexibility enables us to adapt the synthetic matrix array to the size of the imaging target area, facilitating versatile adjustments based on the specific imaging requirements. It is crucial for future implementation, especially for clinical applications.

However, due to mechanically scanning, our system is not suitable to real-time 3D PA imaging. The scanning time for 3D imaging in this work is around 5 min, posing a significant limitation to achieving dynamic 3D visualization. This sets a strict constraint of our system, as it is only suitable to image body parts with less motion. Additionally, with a wide illumination requirement, our method demands much higher laser energy compared to focused arrays.

Future work is needed to improve the performance. First, due to the very low delivery efficiency of glass fiber bundle, the excitation laser power is very limited. We will replace it with high efficiency one to double or even triple the power, which will effectively increase both signal to noise ratio and imaging depth. In addition, replacing a laser system with higher power also helps. But considering the availability and operation feasibility of high-power laser systems in practical use, the laser fluence can only be enhanced about 10 times. Second, to enhance the imaging speed, we plan to use multiple 1D unfocused arrays that are evenly intermittently aligned to work in parallel, which will increase the system cost. For better spatial resolution, unfocused 1D arrays with higher frequency will be explored. Besides system development, we will also explore imaging other valuable body parts, such as the foot, as well as using more wavelengths to perform functional PA imaging.

## 5 Appendix: Supplementary Material

Video 1. 3D animation of the reconstructed vessels (MP4, 1.27 MB [URL: https://doi.org/10.1117/1.JBO.29.S1.S11519.s1]).

## Data Availability

The data utilized in this study are available from the authors upon request.
